# Spatial distribution and driving factors of the associations between temperature and influenza-like illness in the United States: a time-stratified case-crossover study

**DOI:** 10.1186/s12889-023-16240-3

**Published:** 2023-07-20

**Authors:** Yongli Yang, Jiao Lian, Xiaocan Jia, Tianrun Wang, Jingwen Fan, Chaojun Yang, Yuping Wang, Junzhe Bao

**Affiliations:** 1 Department of Epidemiology and Biostatistics, College of Public Health, Zhengzhou University, Zhengzhou, 450001 Henan China; 2 School of Public Health, Jilin University, Changchun, 130021 Jilin China

**Keywords:** Influenza-like illness, Time-stratified case-crossover study, Spatial distribution, Attributable fraction, Driving factors

## Abstract

**Background:**

Several previous studies investigated the associations between temperature and influenza in a single city or region without a national picture. The attributable risk of influenza due to temperature and the corresponding driving factors were unclear. This study aimed to evaluate the spatial distribution characteristics of attributable risk of Influenza-like illness (ILI) caused by adverse temperatures and explore the related driving factors in the United States.

**Methods:**

ILI, meteorological factors, and PM_2.5_ of 48 states in the United States were collected during 2011–2019. The time-stratified case-crossover design with a distributed lag non-linear model was carried out to evaluate the association between temperature and ILI at the state level. The multivariate meta-analysis was performed to obtain the combined effects at the national level. The attributable fraction (AF) was calculated to assess the ILI burden ascribed to adverse temperatures. The ordinary least square model (OLS), spatial lag model (SLM), and spatial error model (SEM) were utilized to identify driving factors.

**Results:**

A total of 7,716,115 ILI cases were included in this study. Overall, the temperature was negatively associated with ILI risk, and lower temperature gave rise to a higher risk of ILI. AF ascribed to adverse temperatures differed across states, from 49.44% (95% eCI: 36.47% ~ 58.68%) in Montana to 6.51% (95% eCI: -6.49% ~ 16.46%) in Wisconsin. At the national level, 29.08% (95% eCI: 27.60% ~ 30.24%) of ILI was attributable to cold. Per 10,000 dollars increase in per-capita income was associated with the increment in AF (OLS: *β* = -6.110, *P* = 0.021; SLM: *β* = -5.496, *P* = 0.022; SEM: *β* = -6.150, *P* = 0.022).

**Conclusion:**

The cold could enhance the risk of ILI and result in a considerable proportion of ILI disease burden. The ILI burden attributed to cold varied across states and was higher in those states with lower economic status. Targeted prevention programs should be considered to lower the burden of influenza.

**Supplementary Information:**

The online version contains supplementary material available at 10.1186/s12889-023-16240-3.

## Background

Influenza is a common respiratory disease caused by the influenza virus. It spreads mainly from person to person via airborne droplets and direct contact and has the characteristics of strong infectivity and rapid transmission [1]. According to the estimation from World Health Organization, there were 290,000 to 650,000 influenza-associated deaths per year worldwide [2]. In the United States (US), the estimated influenza burden was 36 million influenza-related illnesses, 16 million influenza-related medical visits, 390,000 influenza-related hospitalizations, and 25,000 influenza-related deaths for 2019–2020 [3]. Therefore, influenza has been a significant public health issue.

Previous studies found that meteorological factors could exert a key influence on influenza activity, especially temperature [4, 5]. A study from Netherlands demonstrated lower temperature was statistically associated with a higher weekly ILI incidence rate [6]. Li Y et al. [7] observed that the association between temperature and influenza presented an approximate “S” shape in Wuhan, China. The hot and cold were both associated with influenza. A study by Carlos R. Oliveira et al. [8] showed no association between temperature and ILI in Salvador, Brazil. Prior studies focused on assessing the association between temperature and influenza in terms of relative risk, with few estimates of the attributable risk due to temperature, such as attributable fraction (AF) and attributable number. Relative risk only offered the impact of a specific temperature on influenza, which presented restricted information on the actual influence of temperature. The AF assessed the proportion of cases of a disease that would not have occurred absent exposure, which better reflected the overall disease burden and disease prevention effectiveness [9]. It was crucial in formulating programs and evaluating the effect of influenza prevention.

Previous studies were mainly performed in a single city or region using various analytical approaches and different model settings, limiting their results' comparability [7, 8, 10]. It did not facilitate further exploration of the differences and drivers of the associations between temperature and influenza. The discrepancies in previous research might result from differences in socioeconomic, demographic, and climatic characteristics [11]. Large-scale multicenter studies in multiple climatic regions or the whole country could provide more reliable evidence about diversity. A recent nationwide survey from China among 30 cities demonstrated that daily mean temperature and the daily influenza incidence showed an N-shaped curve with the peak risk temperature at 5.1℃. People living in south China were more vulnerable to sensitive ambient temperatures of 1.6℃–14.4℃ [12]. Presently, the attributable risk of influenza caused by adverse temperatures and the corresponding driving factors are unclear.

In this study, we aimed to evaluate the effects of temperature on ILI in terms of relative risk and attributable risk across 48 contiguous US states; we further explored spatial distribution and driving factors of the effects. It could provide epidemiological evidence for developing targeted influenza prevention and control strategies and lower the burden of influenza.

## Methods

### Data collection

We collected the data of ILI, outpatient visits and the percentage of influenza-like cases (ILI% = the number of influenza-like cases/the number of outpatient visits × 100%) for 48 contiguous states in the US from the 1st week of 2011 to the 52nd week of 2019 (1 January 2011 to 31 December 2019) from the Centers for Disease Control and Prevention (CDC, https://www.cdc.gov/flu/weekly), which tracked ILI through the Outpatient Influenza-like Illness Surveillance Network (ILI-Net). Florida was ruled out due to the lack of available data on ILI. ILI was defined as “fever (temperature of 100 F (37.8℃) or greater) plus a cough and/or a sore throat without a known cause other than influenza”.

Meteorological data were downloaded from National Oceanic & Atmospheric Administration (NOAA, https://www.ncei.noaa.gov), consisting of daily mean temperature (°C), relative humidity (%), air pressure (kpa), wind speed (m/s), and daily cumulative precipitation (mm). Daily mean PM_2.5_ concentration (μg/m^3^) data were derived from the Environmental Protection Agency (EPA, https://www.epa.gov/outdoor-air-quality-data). For each state, three monitor stations were selected from the north (east), central, and south (west), and values on the above factors from them were averaged to generate estimates for each state. Considering that ILI data were reported in weekly time units, we calculated the weekly averages of meteorological factors (except precipitation) and PM_2.5_ and weekly cumulative precipitation.

Demographic and economic data, including the percentage of females, percentage of people under 5 years old, population, and per-capita income (in the past 12 months) for each state in US, were collected. The population was obtained from the 2010 and 2020 US Census (https://www.census.gov), and the mean value of 2010 and 2020 was adopted. Other factors were obtained from the American Community Survey (ACS, https://www.census.gov/programs-surveys/acs) during 2011–2019, and their nine-year average values were used.

### Statistical analysis

#### First stage analysis: estimating the state-specific association

Time-stratified case-crossover design with a distributed lag nonlinear model (DLNM) based on a conditional quasi-Poisson regression model was conducted to evaluate the association between temperature and ILI for each state. The "case-crossover design" is often used for individual data, and the analysis is performed using conditional logistic regression [13, 14]. Additionally, this design can also be used for aggregate data (e.g., number of incidences per day), and the analysis can also be conducted using conditional Poisson (or quasi-Poisson) regression in addition to conditional logistic regression [14–17]. Armstrong et al. compared conditional Poisson (including quasi-Poisson) and conditional logistic regression and found similar results for both analysis methods, while conditional quasi-Poisson regression can adjust for overdispersion and autocorrelation [15]. Previously the time-stratified case-crossover design combined with a conditional quasi-Poisson regression model was mainly used for daily time series data, where the strata were matched days based on the same day of the week, calendar month, and year. Control days were selected from the same day of the week, within the same calendar month and year [15–17]. The data in this study was weekly time series data, which cannot control for the "day of the week" effect. Since the government reported 52 weeks of results each year, we divided 4 consecutive weeks as a group (here, groups were similar to months, and there were 12 months but 13 groups per year). Therefore, control weeks in this study were selected from the same group, within the same year.

The DLNM examined a nonlinear exposure–response association and a lag-response association by cross-basis function [18]. We fitted the exposure–response association using a natural cubic B-spline with 3 degrees of freedom (df) and the lag-response association using a natural cubic B-spline. To fully consider the delayed effects and harvesting effects of temperature, a maximum lag of 3 weeks was adopted [19]. Furthermore, models included natural cubic B spline with 3 df of relative humidity and PM_2.5_, the categorical variable of vacation as confounders, and log-transformed outpatient visits as an offset.

For each state, based on the cumulative temperature–ILI associations from the above model, we reported the relative risk with 95% confidence interval (CI) for extreme cold (5th percentile of the temperature distribution), with the median of the temperature distribution as the reference. The number and fraction of ILI attributable to cold (below the median of temperature) were estimated using a previously described method [20, 21]. In brief, for each state, the cumulative relative risk corresponding to each week’s temperature was used to calculate the attributable number and AF. The attributable number of ILI caused by cold was calculated by adding the subsets of weeks with corresponding temperature (below the median of temperature) and its ratio with the number of ILI provided the AF. The 95% empirical confidence interval (eCI) for AF were calculated using Monte Carlo simulations.

#### Second stage analysis: pooling the state-specific estimates

The state-specific associations from the first stage analysis were combined to estimate the pooled effects of temperature on ILI at the national level using multivariate meta-analysis based on a random-effect model, as applied by previous studies [22, 23]. At the national level, the mean of the 5th percentile of temperature across all states was defined as extreme cold. And the mean of the 50th percentile of temperature distributions for all states was defined as the reference. Likewise, the relative risk with 95% CI for extreme cold was reported. The national AF was calculated by the ratio of the sum of the attributable number for all states to the total ILI cases.

#### Third stage analysis: exploring the driving factors of AF distribution

Global spatial autocorrelation was utilized to estimate the spatial correlation between AF of ILI due to cold, with global Moran's I as an indicator [24]. The statistically significant value of Moran's I indicates spatial autocorrelation. The greater the absolute value, the stronger the spatial autocorrelation [25]. To explore the impact of demographic, climatic, and economic factors on AF, non-spatial (ordinary least square model, OLS) and spatial regression models (spatial lag model and spatial error model, SLM and SEM) were carried out. The OLS model was applied to assess the relationship between driving factors and AF without considering spatial dependence. SLM and SEM could better investigate the relationship between AF and driving factors by describing spatial dependence as a lag or error term, respectively. SLM assumed that the AF values influenced each other in neighboring areas, and SEM assumed that the spatial dependence of OLS residuals was derived from the error, which might be due to the spatial dependence of neighboring areas [24, 26].

Firstly, we fitted univariate models with state-specific characteristics, including the percentage of females, percentage of people under 5 years old, population, per-capita income, annual mean temperature, the mean temperature in winter (December-February), and annual relative humidity one at a time. And then the multivariate model was fit with statistically significant (*P* < 0.05) variables identified by the univariate models.

### Sensitivity analysis

Sensitivity analyses were performed to assess the stability of our results. First, we changed the df values for relative humidity and PM_2.5_ from 2 to 4. Second, we also repeated the analysis after excluding some states (Idaho, Delaware, Montana, Iowa, and North Dakota), where the number of weeks with zero counts of ILI accounted for more than one-eighth of the total weeks from 2011 to 2019. Third, the natural cubic B spline with 3 df of air pressure (kpa), wind speed (m/s), and precipitation (mm) were also included in the model.

### Statistical analysis software

Estimating the state-specific association and pooling the state-specific estimates were performed using R software (version 4.0.5), with the ‘dlnm’ package to fit DLNM and the ‘mvmeta’ package to conduct the multivariate meta-analysis. Global spatial autocorrelation analysis, OLS, SLM, and SEM were conducted by GeoDa 1.18.0. A two-tailed *P* < 0.05 was considered statistically significant.

## Results

### Descriptive analysis

**Fig. 1:**
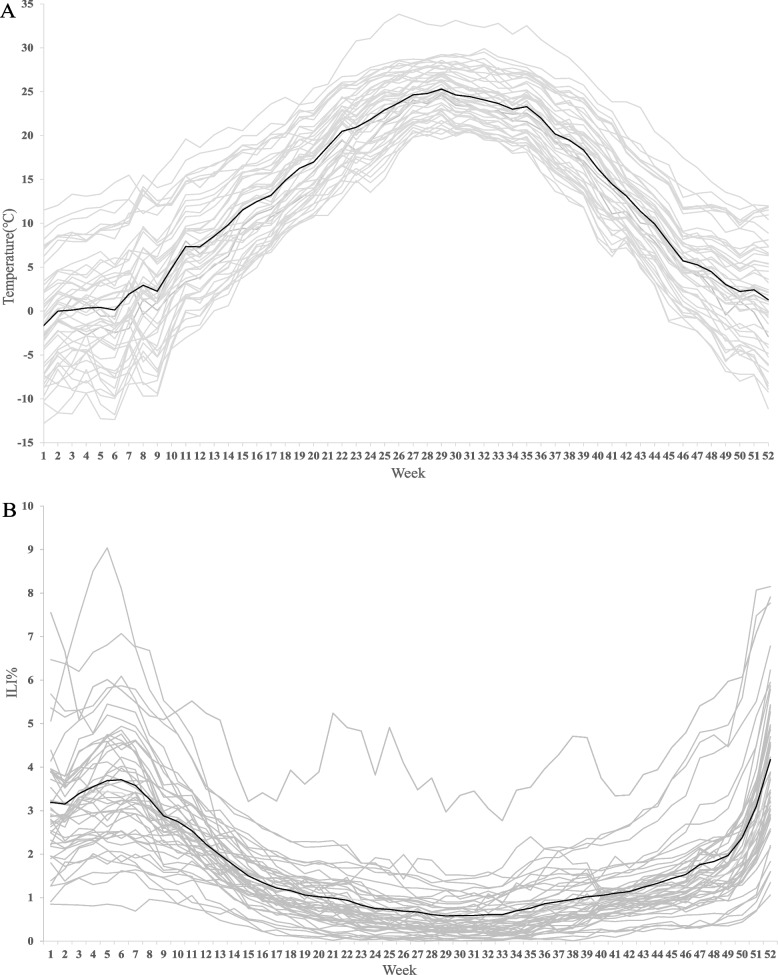
The spatial–temporal distribution of weekly mean temperature and ILI% for 48 states in the US. **A** distribution of weekly mean temperatures, **B** distribution of weekly mean ILI%. Black solid curve and grey dotted lines correspond to nation and 48 states, respectively. ILI%, influenza like-illness cases / outpatient visits*100%

This study included 7,716,115 ILI cases in the US between 2011 and 2019. During the study period, the cumulative number of ILI was higher in Virginia (*N* = 943,247), Louisiana (*N* = 671,171), and Georgia (*N* = 580,205). In contrast, the cumulative number of ILI was lower in Delaware (*N* = 7,785), Montana (*N* = 9,642), and New Hampshire (*N* = 9,997) (Table 1). A considerable variation in meteorological factors and PM_2.5_ was found among these states. It ranged from 5.86 °C to 20.33 °C for the temperature, 39.37% over 73.24% in for relative humidity, and 4.81ug/m^3^ over 10.2ug/m^3^ for PM_2.5_. Figure 1A and B showed the distribution of temperature and ILI% at different times. The ILI% in weeks with low temperature was higher than in other weeks with high temperature.

**Table 1:** State-specific summary statistics for ILI, meteorological factors and PM_2.5_ for 48 states in the US from 2011 to 2019

State	ILI (*N*)	ILI%	Temperature (°C)	Relative humidity (%)	PM_2.5_ (ug/m^3^)
	Cases	Mean (SD)	Mean (SD)	Mean (SD)	Mean (SD)
Alabama	328,429	3.28(2.74)	18.59 (7.52)	71.81 (6.90)	8.75(2.62)
Arizona	187,864	1.80(1.18)	18.05 (7.78)	39.37 (13.91)	6.90(2.63)
Arkansas	43,745	2.00(2.27)	16.91 (8.53)	71.11 (7.61)	9.20(2.94)
California	463,664	2.28(1.08)	18.61 (6.45)	54.26 (12.29)	9.75(4.91)
Colorado	152,504	1.26(1.25)	11.22 (9.39)	53.83 (10.88)	6.42(2.5)
Connecticut	45,105	1.50(1.49)	11.00 (9.49)	68.58 (8.03)	7.49(2.81)
Delaware	7,785	0.52(0.92)	14.10 (8.98)	69.28 (7.77)	7.90(2.96)
District of Columbia	83,283	4.61(2.68)	15.79 (9.08)	62.68 (8.64)	8.98(2.91)
Georgia	580,205	2.17(2.00)	18.6 (7.26)	72.66 (7.10)	9.20(2.96)
Idaho	19,085	1.29(1.35)	6.95 (8.99)	61.59 (13.79)	10.04(7.59)
Illinois	468,649	1.95(1.14)	12.28 (10.28)	71.03 (6.84)	9.31(2.79)
Indiana	54,682	1.61(1.59)	12.42 (9.96)	70.43 (6.67)	9.58(3.62)
Iowa	18,445	0.66(0.85)	9.93 (11.4)	71.64 (7.6)	8.06(3.06)
Kansas	72,742	1.67(2.09)	13.34 (10.34)	64.13 (8.92)	8.22(3.51)
Kentucky	90,667	1.22(2.14)	14.53 (8.92)	69.46 (7.58)	9.60(3.37)
Louisiana	671,171	2.96(2.13)	20.33 (7.23)	73.24 (7.00)	9.20(2.83)
Maine	30,946	0.91(0.64)	6.24 (10.7)	71.3 (7.83)	6.21(2.07)
Maryland	79,692	1.89(1.39)	14.04 (9.08)	67.61 (7.99)	8.56(2.99)
Massachusetts	203,981	1.20(0.78)	9.73 (9.54)	68.66 (7.78)	6.82(2.62)
Michigan	130,665	1.26(0.97)	8.51 (10.43)	72.61 (7.22)	7.11(2.99)
Minnesota	48,301	1.68(1.28)	5.98 (12.2)	69.92 (7.61)	6.32(2.58)
Mississippi	300,997	3.36(2.11)	19.23 (7.48)	71.59 (6.10)	9.75(2.92)
Missouri	52,929	1.62(1.94)	13.41 (9.97)	69.87 (7.89)	9.45(2.93)
Montana	9,642	0.37(0.61)	6.15 (10.67)	62.95 (10.39)	7.84(5.23)
Nebraska	58,725	1.91(1.95)	10.08 (10.53)	65.80 (8.27)	6.53(2.65)
Nevada	85,254	1.17(0.95)	12.38 (8.8)	41.76 (15.82)	7.22(3.79)
New Hampshire	9,997	0.53(0.74)	7.66 (10.21)	70.86 (7.52)	6.21(2.45)
New Jersey	215,507	2.29(1.67)	12.93 (9.22)	67.55 (8.15)	7.76(2.68)
New Mexico	145,470	2.16(1.5)	14.83 (8.9)	42.55 (13.38)	6.91(2.38)
New York	161,668	1.66(1.73)	10.07 (10.06)	68.98 (6.57)	7.67(2.76)
North Carolina	225,984	1.59(1.55)	15.72 (8.05)	70.27 (8.11)	7.65(2.33)
North Dakota	11,697	1.20(1.49)	5.86 (12.14)	69.22 (8.7)	5.51(2.85)
Ohio	81,029	0.95(0.86)	12.22 (9.84)	68.99 (6.79)	9.44(3.19)
Oklahoma	74,113	2.64(2.87)	16.30 (9.50)	62.28 (9.4)	8.22(2.81)
Oregon	115,259	1.11(1.09)	10.29 (7.39)	64.24 (13.06)	9.52(7.52)
Pennsylvania	231,068	1.55(1.14)	11.73 (9.52)	68.62 (7.41)	9.54(3.4)
Rhode Island	23,228	0.77(1.21)	11.34 (8.80)	71.45 (8.16)	6.03(2.21)
South Carolina	69,292	1.58(2.24)	19.00 (7.46)	67.48 (7.86)	8.70(2.79)
South Dakota	55,315	1.23(0.93)	7.81 (11.55)	68.27 (9.00)	7.44(3.36)
Tennessee	94,099	1.55(1.87)	16.22 (8.55)	69.26 (7.35)	9.28(2.77)
Texas	574,351	3.42(2.54)	18.75 (8.00)	61.22 (9.30)	10.20 (2.67)
Utah	162,785	1.71(1.26)	8.77 (9.87)	53.47 (15.37)	6.74(5.14)
Vermont	25,889	1.75(1.13)	7.16 (10.46)	72.96 (7.41)	8.17(4.6)
Virginia	943,247	2.09(1.65)	14.91 (8.56)	66.3 (9.53)	7.91(2.49)
Washington	32,105	0.93(1.05)	10.59 (8.12)	66.31 (15.4)	9.36(6.16)
West Virginia	93,215	1.28(1.41)	12.93 (8.75)	70.02 (7.75)	8.67(3.06)
Wisconsin	54,127	1.33(1.07)	7.27 (11.39)	71.19 (7.49)	7.82(3.22)
Wyoming	27,513	1.13(1.42)	7.57 (10.46)	55.82 (13.91)	4.81(2.89)

*Abbreviations: SD* Standard deviation

### Associations between temperature and ILI

**Fig. 2:**
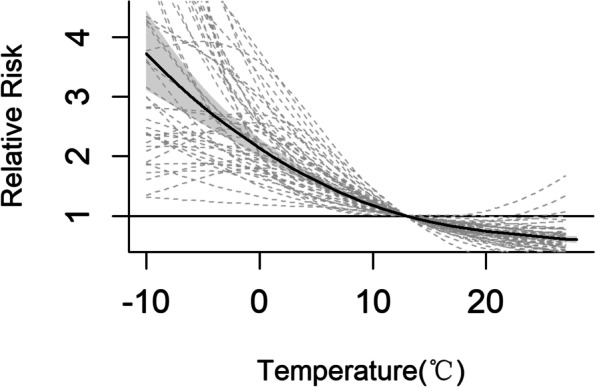
Pooled cumulative exposure–response curves for associations between temperature and ILI at the national level (black solid curve). Grey dotted lines indicate 48 state-specific curves

Figure 2 depicted the pooled cumulative exposure–response curve for the associations between temperature and ILI. From the curve, we observed that the ILI risk increased as the temperature lowered.

Table S1 showed the effects of extreme cold on ILI in each state, and the effects differed among states. The effects were stronger in New Mexico (Relative risk = 4.69, 95% CI: 3.72 ~ 5.92), North Dakota (Relative risk = 3.79, 95% CI: 2.45 ~ 5.85), and Washington (Relative risk = 3.66, 95% CI: 2.62 ~ 5.11).

At the national level, extreme cold (-2.59 °C) was associated with a higher risk of ILI (Relative risk = 2.46, 95% CI: 2.26 ~ 2.68), compared with the reference temperature(12.82 °C). From an attribution risk perspective, the AF of ILI due to cold was 29.08% (95% eCI: 27.60% ~ 30.24%). Accordingly, these days resulted in 2,243,471 (95% eCI: 2,126,231 ~ 2,332,994) ILI cases in this study population. 

### Spatial distributions of AF

**Fig. 3:**
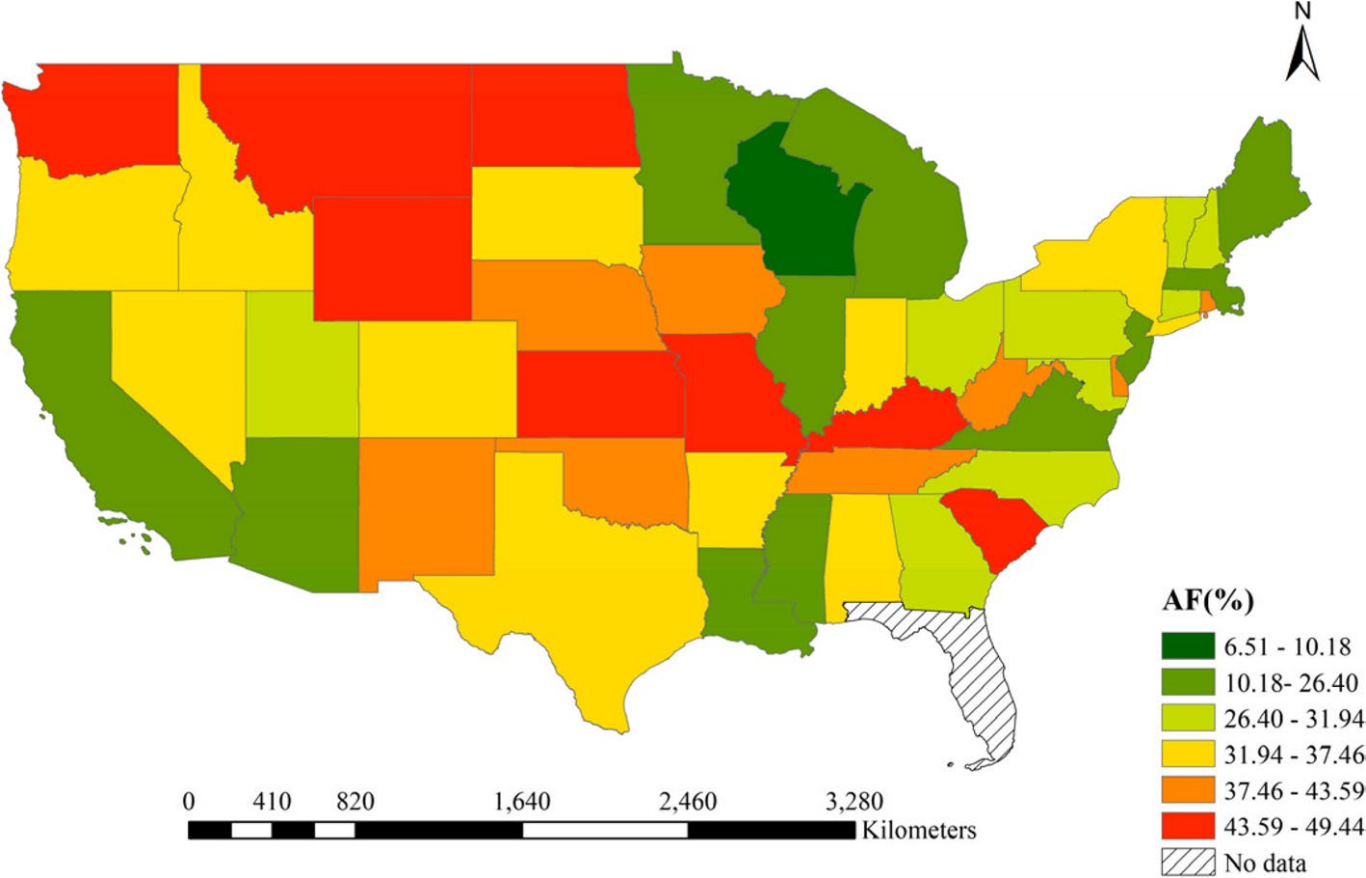
The spatial distribution of AF of ILI ascribed to cold in the US during 2011–2019

Figure 3 displayed the spatial distribution of AF in the US. The AF varied substantially among states. Table S2 reported each state’s attributable number and fraction of ILI associated with cold. The AF in Montana (AF = 49.44%, 95% eCI: 36.47% ~ 58.68%), Kansas (AF = 49.19%, 95% eCI: 40.75% ~ 56.42%) and Wyoming (AF = 47.91%, 95% eCI: 36.13% ~ 57.17%) were relatively high. The AF in District of Columbia (AF = 10.18%, 95% eCI: 2.52% ~ 17.12%) and New Jersey (AF = 19.77%, 95% eCI: 13.82% ~ 25.36%) were relatively low.

### Driving factors of the differences in the spatial distribution of AF

In global spatial autocorrelation analysis, Moran's I was 0.2142 (*P* < 0.05), which showed a positive spatial correlation of AF of ILI caused by cold. The spatial regression models were required. The models with a single factor found that the increment in AF was associated with per 1% increase in the percentage of females (OLS: *β* = -4.737, 95% CI = -8.471 ~ -1.003, *P* = 0.017; SLM: *β* = -3.689, 95% CI = -7.387 ~ 0.009, *P* = 0.051; SEM: *β* = -4.905, 95% CI = -9.452 ~ -0.358, *P* = 0.034) and per 10,000 dollars increase in per-capita income (OLS: *β* = -7.676, 95% CI = -12.719 ~ -2.633, *P* = 0.004; SLM: *β* = -6.573, 95% CI = -11.430 ~ -1.716, *P* = 0.008; SEM: *β* = -7.898, 95% CI = -13.366 ~ -2.430 *P* = 0.005). When per-capita income, percentage of females, and the population were simultaneously incorporated into a multivariate model. Only the impact of per 10,000 dollars increase in per-capita income on AF (OLS: *β* = -6.110, 95% CI = -11.120 ~ -1.100, *P* = 0.021; SLM: *β* = -5.496, 95% CI = -10.192 ~ -0.800, *P* = 0.022; SEM: *β* = -6.150, 95% CI = -11.430 ~ -0.870, *P* = 0.022) was observed (Table 2). For other factors, differences in the AF across states were not statistically associated with them.

**Table 2:** Effects of state-specific factors in non-spatial and spatial regression models

Variables	OLS			SLM			SEM		
	*β*	95% CI	*P*	*β*	95% CI	*P*	*β*	95% CI	*P*
**Single variable model**									
Percentage of female (%)	**-4.737**	**-8.471 ~ -1.003**	**0.017**	**-3.689**	**-7.387 ~ 0.009**	**0.051**	**-4.905**	**-9.452 ~ -0.358**	**0.034**
Percentage of under 5 years (%)	1.878	-2.04 ~ 5.796	0.353	0.643	-2.93 ~ 4.216	0.724	-0.362	-4.807 ~ 4.083	0.873
Population (10,000 persons)	-0.031	-0.072 ~ 0.010	0.146	-0.030	-0.065 ~ 0.005	0.096	-0.026	-0.059 ~ 0.007	0.143
Mean temperature (℃)	-0.123	-0.811 ~ 0.565	0.728	-0.131	-0.752 ~ 0.49	0.679	-0.196	-1.121 ~ 0.729	0.678
Mean temperature of winter (℃)	-0.082	-0.580 ~ 0.416	0.748	-0.085	-0.536 ~ 0.366	0.711	-0.142	-0.818 ~ 0.534	0.680
Mean Relative humidity (%)	-0.052	-0.387 ~ 0.283	0.762	-0.019	-0.321 ~ 0.283	0.903	-0.014	-0.443 ~ 0.415	0.948
Per-capita income (10,000 dollars)	**-7.676**	**-12.719 ~ -2.633**	**0.004**	**-6.573**	**-11.430 ~ -1.716**	**0.008**	**-7.898**	**-13.366 ~ -2.430**	**0.005**
**Multiple variable model**									
Percentage of female (%)	-3.200	-6.824 ~ 0.424	0.090	-2.397	-5.950 ~ 1.156	0.186	-3.655	-7.832 ~ 0.522	0.086
Per-capita income (10,000 dollars)	**-6.110**	**-11.120 ~ -1.100**	**0.021**	**-5.496**	**-10.192 ~ -0.800**	**0.022**	**-6.150**	**-11.430 ~ -0.870**	**0.022**
Population (10,000 persons)	-0.033	-0.068 ~ 0.002	0.081	-0.033	-0.066 ~ 0.001	0.055	-0.028	-0.061 ~ 0.005	0.096

### Sensitivity analysis

Sensitivity analysis showed that our models and results were stable and reliable (Table S3).

## Discussion

Based on 7,716,115 ILI cases across 48 states in the US, this study characterized the associations between temperature and ILI and found that cold was associated with a higher risk of ILI. In total, 29.08% of ILI could be attributed to cold, a considerable value at the national level. Moreover, AF varied across states, with Montana (AF = 49.44%, 95% eCI: 36.47% ~ 58.68%) higher than other states. In addition, it was found that economic status was the main driving factor, and people living in lower economic states were sensitive to cold.

Our result of the association between cold and ILI coincided with most previous studies. Zhang Y et al. [27] showed that low temperatures led to more influenza cases in Shanghai. Peci A et al. [28] found a negative association of temperature with influenza virus infections that low temperature enhanced influenza activity in Toronto, Ontario, Canada. There were some hypotheses in favor of explaining the phenomenon that low temperatures increased the risk of influenza. Firstly, low temperatures may prolong the survival of the influenza virus and make more people stay indoors, increasing the chances of influenza virus infections [29, 30]. Secondly, inhalation of cold air causes cooling of the nasal epithelium, which could increase the viscosity of the mucous layer and reduce mucociliary clearance. It is favorable to viral spread within the respiratory tract [31]. Finally, low temperatures may affect host immunity, which makes the host more vulnerable to the influenza virus [32].

On the other hand, several studies found no relationship between cold and influenza. Steven Yuk-Fai Lau et al. [33] suggested that low temperature was not linked to influenza infections in Hangzhou, China. A multicenter study from 45 prefectures in Japan observed the relationship between lower temperatures and higher ILI risks in about 65% of the prefectures. At the same time, the cold was not related to ILI in several areas [34]. This study found no association between cold and ILI in Wisconsin. Therefore, the multicenter research can comprehensively find the association characteristics between ambient temperature and ILI. In addition, some studies found that high temperatures positively impact the activity of influenza. Dai Q et al. [35] observed that low temperatures and high temperatures enhanced the risk of ILI and influenza in Jiangsu Province, China. However, the mechanism remained unclear. Apart from statistical methods and model parameters, heterogeneity of the relationships between temperature and influenza activity might arise from differences in various characteristics, such as humidity, rainfall, income, and health resources [36, 37].

DLNM has recently been widely applied to investigate the relationship between temperature and influenza activity [12, 38]. It adjusted the seasonal and long-term trends by incorporating the smooth function for the “time” in the model. Most studies chose 7 df per year for the “time” or based on information criteria (such as Quasi Akaike information criteria) to choose df, mainly used for daily time series data. For studies with weekly time series data, the choice of df values for the “time” was yet to be a consensus [39–41]. The time-stratified case-crossover design combined with quasi-Poisson regression could control long-term trends and seasonality, and adjust for overdispersion and autocorrelation [15, 16, 42].

Little knowledge was available about the disease burden of ILI attributable to cold. Our results showed that 29.08% (95% eCI: 27.60% ~ 30.24%) of ILI was caused by cold. A study conducted among 30 cities in China over the period 2016–2019 found 60% (95% eCI: 54.3% ~ 64.3%) of influenza incidence was attributed to ambient temperature during the days with sensitive temperatures (1.6℃–14.4℃) [12]. Differences in attributed risk in the two studies might be due to the diversity of socioeconomic and demographic characteristics and the difference in analytical approaches [43, 44]. The burden of ILI caused by cold cannot be ignored, which is vital for clinicians and public health officials who advise the public.

Our results showed greater attributable burdens caused by cold could be observed in states with lower economic status. Many studies have demonstrated that the population in poorer areas always had a higher risk of influenza outcomes (infection, hospitalizations, and mortality) [45, 46]. The underlying mechanisms for the effect of the economy remained unclear, which might be explained by the following reasons: individuals with low economic status increased exposure to low temperatures indoors and outside [47]. In contrast, people with high economic status could better afford health-protecting behaviors [48]. Our findings highlighted that influenza prevention and protection measures should be implemented in poorer areas.

In the relationship between temperature and ILI, the cold might increase the risk of ILI. However, no driving effect of temperature on the distribution of AF was found. For a specific state, the AF was computed from the cumulative relative risk for each week's temperature, so the lower temperature, the higher AF. On the other hand, the health effects of temperature were not only influenced by the temperature of the region but may also be influenced by other factors such as population adaptability and economy in the region [49–51]. Renjie Chen et al. [50] found that the mortality burden attributable to cold was more prominent in southern cities, while those for hot temperatures were in the opposite direction. Wenjuan Ma et al. [51] observed larger cold effects in southern cities and larger hot effects in northern cities. This phenomenon may be related to central heating in northern cities and the higher adaptability of the people in northern cities to cold than those in southern cities. Therefore, in a nationwide study, some states may have lower average temperatures, but the AF caused by low temperatures was not necessarily high due to factors such as high adaptive capacity and economic level, ultimately resulting in temperatures that do not effectively drive differences in the spatial distribution of AF values.

There are several merits to this study. Firstly, 9 years of long-term time-series data and a relatively large sample size across 48 states in the US were applied, which helped enhance the reliability and representativeness of the findings. Secondly, we estimated the relative risk of ILI associated with extreme cold and the attributable fractions caused by cold at the state and national levels to provide novel evidence. Thirdly, this study filled the gap in evidence regarding spatial variation and driving factors of the association between temperature and ILI in the US.

Several potential limitations need to be noted in this study. Firstly, since only state-level summary data on ILI was available, we used state-level ambient temperature from outdoor monitors as exposure. It might give rise to exposure measurement errors. Secondly, this study used outdoor meteorological data without considering the impact of indoor meteorological conditions, which may affect flu virus transmission. Thirdly, influenza vaccination might reduce reportable flu-like cases, illness severity, and possible deaths. However, due to data limitations, this study did not consider the effects of influenza vaccines on temperature effects. Fourthly, we only adjusted PM_2.5_ and not adjusted other pollutants (such as CO and NO_2_) due to the high proportion of missing data.

## Conclusions

In summary, this nationwide study indicated that cold could enhance the risk of ILI and result in a considerable ILI disease burden. The distribution of the AF ascribed to cold differed across states of the US, and it was higher in the states with lower economic status. Our findings provide evidence for developing targeted influenza prevention programs and lowering the influenza burden.

## Supplementary Information

**Additional file 1.**

## Data Availability

The data used in this study are openly available in the Centers for Disease Control and Prevention (https://www.cdc.gov/flu/weekly/), National Oceanic & Atmospheric Administration (https://www.ncei.noaa.gov), Environmental Protection Agency (https://www.epa.gov/outdoor-air-quality-data), US Census (https://www.census.gov), and American Community Survey (https://www.census.gov/programs-surveys/acs).

## Abbreviations

US: United States
ILI: Influenza-like illness
ILI-Net: Influenza-like Illness Surveillance Network
AF: Attributable fraction
DF: Degree of freedom
DLNM: Distributed lag nonlinear model
OLS: Ordinary least square model
SLM: Spatial lag model
SEM: Spatial error model
eCI: Empirical confidence interval
