# Supplementary material for: Spatial distribution and driving factors of the associations between temperature and influenza-like illness in the United States: a time-stratified case-crossover study
Source: BMC Public Health. 2023 Jul 20;23:1403. doi: 10.1186/s12889-023-16240-3 (PMC10360314; doi:10.1186/s12889-023-16240-3)
Supplement: Supplementary file 1 — Additional file 1. [file 12889_2023_16240_MOESM1_ESM.docx]

Table S1. State-specific relative risk of ILI associated with extreme temperature

| State | Relative risk | 95% Confidence interval |
| --- | --- | --- |
| Alabama | 2.98 | 2.47~3.59 |
| Arizona | 1.81 | 1.53~2.13 |
| Arkansas | 2.64 | 1.92~3.63 |
| California | 1.98 | 1.70~2.31 |
| Colorado | 2.79 | 2.23~3.5 |
| Connecticut | 1.84 | 1.48~2.28 |
| Delaware | 1.85 | 1.09~3.15 |
| District of Columbia | 1.35 | 1.07~1.71 |
| Georgia | 2.63 | 2.22~3.11 |
| Idaho | 2.65 | 1.87~3.76 |
| Illinois | 1.92 | 1.61~2.28 |
| Indiana | 2.34 | 1.75~3.14 |
| Iowa | 2.29 | 1.6~3.26 |
| Kansas | 3.12 | 2.38~4.10 |
| Kentucky | 2.71 | 1.90~3.86 |
| Louisiana | 2.34 | 1.99~2.75 |
| Maine | 1.68 | 1.27~2.22 |
| Maryland | 2.37 | 1.77~3.18 |
| Massachusetts | 1.75 | 1.47~2.09 |
| Michigan | 1.66 | 1.31~2.11 |
| Minnesota | 1.60 | 1.21~2.13 |
| Mississippi | 1.97 | 1.68~2.32 |
| Missouri | 2.76 | 2.06~3.70 |
| Montana | 3.53 | 2.28~5.46 |
| Nebraska | 3.01 | 2.20~4.13 |
| Nevada | 2.90 | 2.30~3.66 |
| New Hampshire | 1.63 | 1.05~2.54 |
| New Jersey | 1.63 | 1.38~1.93 |
| New Mexico | 4.69 | 3.72~5.92 |
| New York | 2.02 | 1.58~2.58 |
| North Carolina | 2.01 | 1.58~2.56 |
| North Dakota | 3.79 | 2.45~5.85 |
| Ohio | 1.88 | 1.43~2.48 |
| Oklahoma | 3.01 | 2.33~3.90 |
| Oregon | 2.46 | 2.03~2.97 |
| Pennsylvania | 1.95 | 1.61~2.35 |
| Rhode Island | 2.02 | 1.40~2.93 |
| South Carolina | 3.17 | 2.45~4.09 |
| South Dakota | 2.20 | 1.79~2.72 |
| Tennessee | 3.06 | 2.14~4.38 |
| Texas | 2.52 | 2.08~3.05 |
| Utah | 2.96 | 2.29~3.83 |
| Vermont | 1.89 | 1.47~2.43 |
| Virginia | 1.56 | 1.29~1.88 |
| Washington | 3.66 | 2.62~5.11 |
| West Virginia | 2.27 | 1.66~3.13 |
| Wisconsin | 1.14 | 0.91~1.43 |
| Wyoming | 3.42 | 2.38~4.94 |

Table S2 Fraction and number of ILI attributable to cold for each state

| State | Attributable fraction (%) | Attributable number (*N*) |
| --- | --- | --- |
| Alabama | 35.63(30.37~41.27) | 117,019(99,754~135,554) |
| Arizona | 22.61(17.75~26.77) | 42,468(33,342~50,294) |
| Arkansas | 37.46(22.84~47.82) | 16,387(9,993~20,921) |
| California | 20.00(15.42~24.25) | 92,751(71,475~112,437) |
| Colorado | 32.81(26.64~38.01) | 50,040(40,623~57,972) |
| Connecticut | 29.92(21.57~36.56) | 13,497(9,728~16,491) |
| Delaware | 39.36(9.21~57.71) | 3,064(717~4,493) |
| District of Columbia | 10.18(2.52~17.12) | 8,480(2,098~14,258) |
| Georgia | 31.01(26.36~35.49) | 179,922(152950~205912) |
| Idaho | 35.00(24.39~44.03) | 6,681(4,655~8,403) |
| Illinois | 25.12(19.21~30.35) | 117,712(90,014~142,217) |
| Indiana | 35.31(24.59~43.5) | 19,310(13,448~23,786) |
| Iowa | 39.00(26.07~48.47) | 7,193(4,809~8,940) |
| Kansas | 49.19(40.75~56.42) | 35,780(29,642~41,042) |
| Kentucky | 44.96(31.82~55.44) | 40,765(28,846~50,262) |
| Louisiana | 26.40(21.41~30.79) | 177,205(143,665~206,659) |
| Maine | 24.02(14.19~32.38) | 7,433(4,390~10,019) |
| Maryland | 31.12(21.94~38.56) | 24,797(17,485~30,726) |
| Massachusetts | 21.98(15.48~27.41) | 44,836(31,582~55,910) |
| Michigan | 24.79(15.56~32.43) | 32,393(20,332~42,377) |
| Minnesota | 23.05(11.46~31.71) | 11,135(5,534~15,317) |
| Mississippi | 22.83(17.26~27.67) | 68,703(51,943~83,275) |
| Missouri | 47.47(38.6~54.86) | 25,124(20,432~29,038) |
| Montana | 49.44(36.47~58.68) | 4,767(3,517~5,657) |
| Nebraska | 43.59(33.86~51.05) | 25,596(19,884~29,978) |
| Nevada | 36.33(29.57~42.16) | 30,976(25,208~35,940) |
| New Hampshire | 30.31(9.72~43.85) | 3,030(971~4,383) |
| New Jersey | 19.77(13.82~25.36) | 42,608(29,793~54,645) |
| New Mexico | 40.70(34.43~45.65) | 59,208(50,082~66,405) |
| New York | 35.62(25.86~43.05) | 57,591(41,815~69,599) |
| North Carolina | 31.94(22.50~39.64) | 72,178(50,851~89,574) |
| North Dakota | 44.78(29.90~55.20) | 5,238(3,498~6,457) |
| Ohio | 30.12(20.14~39.09) | 24,403(16,323~31,676) |
| Oklahoma | 42.99(34.31~50.00) | 31,858(25,430~37,058) |
| Oregon | 35.23(28.14~40.60) | 40,601(32,435~46,792) |
| Pennsylvania | 28.46(21.20~34.20) | 65,753(48,997~79,019) |
| Rhode Island | 43.35(26.59~55.40) | 10,068(6,175~12,868) |
| South Carolina | 44.81(36.20~51.46) | 31,049(25,082~35,659) |
| South Dakota | 34.62(27.49~40.62) | 19,149(15,207~22,472) |
| Tennessee | 41.03(28.76~50.21) | 38,605(27,063~47,249) |
| Texas | 33.04(27.36~37.6) | 189,783(157,162~215,952) |
| Utah | 31.44(25.25~36.56) | 51,184(41,096~59,506) |
| Vermont | 27.46(17.26~35.71) | 7,109(4,469~9,246) |
| Virginia | 23.48(15.63~29.48) | 221,434(147,465~278,081) |
| Washington | 46.67(36.43~54.41) | 14,984(11,697~17,469) |
| West Virginia | 39.59(27.85~49.12) | 36,904(25,956~45,792) |
| Wisconsin | 6.51(-6.49~16.46) | 3,522(-3,513~8,910) |
| Wyoming | 47.91(36.13~57.17) | 13,180(9,941~15,730) |

Table S3 Sensitivity analysis results for different models

|  | Relative risk | Attributable fraction (%) |
| --- | --- | --- |
| DF for Relative humidity:2 ^a^ | 2.47(2.27~2.69) | 29.21(27.59~30.44) |
| DF for Relative humidity:4 ^a^ | 2.46(2.26~2.68) | 29.08(27.60~30.15) |
| DF for PM_2.5_: 2 ^b^ | 2.47(2.27~2.70) | 29.13(27.64~30.32) |
| DF for PM_2.5_: 4 ^b^ | 2.45(2.25~2.66) | 28.94(27.41~30.15) |
| Excluded data ^c^ | 2.40(2.20~2.62) | 28.98(27.52~30.20) |
| Adjustment ^d^ | 2.49(2.28~2.71) | 29.40(27.90~30.53) |

Abbreviations: DF, degrees of freedom

^a^ the df for relative humidity were changed from 2 to 4.

^b^ the df for PM_2.5_ were changed from 2 to 4.

^c^ Idaho, Delaware, Montana, Iowa, and North Dakota were excluded.

^d^ the natural cubic B spline with 3 df of air pressure (kpa), wind speed (m/s) and precipitation (mm) were included in model
